# The modulation of pulmonary group 2 innate lymphoid cell function in asthma: from inflammatory mediators to environmental and metabolic factors

**DOI:** 10.1038/s12276-023-01021-0

**Published:** 2023-09-11

**Authors:** Christina Li-Ping Thio, Ya-Jen Chang

**Affiliations:** 1 Institute of Biomedical Sciences, Academia Sinica, Taipei City, 115 Taiwan; 2 Institute of Translational Medicine and New Drug Development, China Medical University, Taichung City, 404 Taiwan

**Keywords:** Innate lymphoid cells, Mucosal immunology

## Abstract

A dysregulated type 2 immune response is one of the fundamental causes of allergic asthma. Although Th2 cells are undoubtedly central to the pathogenesis of allergic asthma, the discovery of group 2 innate lymphoid cells (ILC2s) has added another layer of complexity to the etiology of this chronic disease. Through their inherent innate type 2 responses, ILC2s not only contribute to the initiation of airway inflammation but also orchestrate the recruitment and activation of other members of innate and adaptive immunity, further amplifying the inflammatory response. Moreover, ILC2s exhibit substantial cytokine plasticity, as evidenced by their ability to produce type 1- or type 17-associated cytokines under appropriate conditions, underscoring their potential contribution to nonallergic, neutrophilic asthma. Thus, understanding the mechanisms of ILC2 functions is pertinent. In this review, we present an overview of the current knowledge on ILC2s in asthma and the regulatory factors that modulate lung ILC2 functions in various experimental mouse models of asthma and in humans.

## Immunology: examining an enigmatic contributor to asthma

A subset of immune cells discovered in 2010 plays a prominent role in asthma, but the nature of their influence is unclear. T cells are the main drivers of the inflammatory response in asthma, but scientists have learned that another group of immune cells known as ‘group 2 innate lymphoid cells’ (ILC2s) also plays a role. Christina Li-Ping Thio and Ya-Jen Chang at the Academia Sinica, Taipei, Taiwan, have reviewed current knowledge of how ILC2s interact with other cells and tissues to influence asthma pathophysiology. Animal and human data strongly suggest that ILC2s help coordinate the immune response to allergen-induced asthma, although it remains unclear whether these cells are associated with more severe or treatment-resistant asthma. The authors highlight molecular signals that affect ILC2 function, including metabolic and dietary factors that could reduce asthma risk.

## Introduction

Asthma is a chronic respiratory disease characterized by airway hyperreactivity (AHR), chronic lower airway inflammation, and airway remodeling. These pathological changes contribute to the hallmark features of asthma, such as shortness of breath, coughing, and chest tightness. The 2019 Global Burden and Disease study estimated that asthma affects over 260 million people globally^1^. Although asthma affects both children and adults, disease prevalence is higher in children, whereas mortality is higher in adults^2^. Asthma is a heterogeneous disease with varying phenotypes (early-onset allergic asthma, late-onset nonallergic eosinophilic asthma, and late-onset nonallergic noneosinophilic asthma) and distinct endotypes, which can be broadly classified as type 2 (T2)-high or T2-low/non-T2^3,4^. T2-high asthma is characterized by the presence of airway and systemic eosinophilia, whereas T2-low asthma can be either neutrophilic or paucigranulocytic^3^.

The T2-high endotype accounts for 50% of mild-to-moderate asthma and likely a large proportion of patients with severe asthma^5^. Type 2 cytokines and their effector cells, namely, T helper 2 (Th2) cells and group 2 innate lymphoid cells (ILC2s), are etiologically related to this endotype^3^.

Although ILC2s were only discovered slightly more than a decade ago, their importance in the initiation and orchestration of T2-driven asthma has been increasingly recognized. ILC2s belong to the ILC family, which includes four other subsets: cytotoxic natural killer (NK) cells, ILC1s, ILC3s, and lymphoid tissue inducer cells^6^. ILC2s are tissue-resident cells found in various mucosal and nonmucosal tissues, such as the lung, skin, and intestine, where they exhibit tissue-specific heterogeneity^7^. Unlike T cells, ILC2s do not possess antigen-specific receptors; therefore, their activation is mediated by the recognition of epithelial-derived cytokines such as IL-33 and IL-25^8^. These cells are often considered the innate counterparts of adaptive Th2 cells due to similarities in their effector functions and transcription factors that regulate their development. Similar to Th2 cells, ILC2 development, and function are governed by GATA3, RORa, and Bcl11b^9–11^, and upon activation, ILC2s produce type 2 cytokines such as IL-13, IL-5, and, to a lesser extent, IL-4^12^. ILC2s also produce IL-9 and amphiregulin, and the latter is associated with tissue-protective repair responses after influenza virus infection^13,14^. Thus, ILC2s play dual roles in promoting airway inflammation and facilitating lung tissue repair during the resolution phase. In this review, we focus on the contribution of ILC2s to asthma and recent insights into the regulation of ILC2 functions in the lungs.

## ILC2s and asthma

### Preclinical models

Studies of mouse models have revealed important roles for ILC2s in type 2-mediated inflammatory responses. Common allergens or proteases such as *Alternaria alternata*, papain, and house dust mites (HDM) activate ILC2s within hours, and these cells produce copious amounts of IL-13 and IL-5 that contribute to asthma-like features in mice, such as eosinophil infiltration, AHR, airway hypertrophy, and mucus hypersecretion^12,15,16^. In addition to allergens, respiratory viruses such as influenza A and respiratory syncytial virus (RSV) have also been reported to activate lung ILC2s and drive AHR and airway inflammation^17,18^. Respiratory viral infections cause asthma exacerbation, which has been linked to Th2 immune responses^19^. Considering that the ILC2 response is enhanced during infection, these cells may play an important role in viral-induced asthma exacerbation. Type 2 cytokines released by ILC2s act not only as effector molecules that cause lung pathogenesis but also as signaling molecules that activate Th2 cell responses. In particular, IL-13 is an important cytokine that induces dendritic cells (DCs) to enhance the memory Th2 cell response during allergen rechallenge^20^. In this context, ILC2s potentiate type 2 inflammation by linking innate and adaptive type 2 immunity.

A recent effort to map the immune landscape of the lung in a mouse model of steroid-resistant asthma exacerbation revealed that ILC2s were one of the major sources of steroid-resistant IL-4 and IL-13 transcripts^21^. Studies of ILC2 sensitivity toward corticosteroid treatment, which is the mainstay therapy for asthma, showed that thymic stromal lymphopoietin (TSLP)/signal transducer and activator of transcription 5 (STAT5) signaling mediated steroid resistance in ILC2s^22^. Mechanistically, TSLP inhibits corticosteroid-induced apoptosis by inducing the antiapoptotic protein Bcl-xL^22^. The role of the IL-33/ST2 axis, however, is contradictory. While one study showed that IL-33 mediated ILC2 resistance to budesonide in the *A. alternata* model^23^, another reported that dexamethasone suppressed ILC2 activation and subsequent airway inflammation in the IL-33 model^22^.

### Human ILC2s in asthma

A growing number of studies have shown the association between ILC2s and asthma. Significantly increased numbers of ILC2s have been detected in the peripheral blood, sputum, and bronchoalveolar lavage fluid (BALF) of asthmatic patients^24–26^. ILC2s from asthmatic patients also exhibit higher activity and produce more type 2 cytokines than those from healthy individuals^27^. Moreover, the ILC2-regulating cytokines IL-33 and TSLP are increased in the BALF of asthmatic patients^24,28^. Similar to the results of mouse studies, ILC2 numbers and type 2 cytokine production were increased 24 hours after allergen challenge in asthmatic patients, resulting in elevated levels of IL-13 and IL-5 in the airways^29,30^. Studies evaluating the correlation between ILC2 numbers and asthma severity, on the other hand, showed disparate results. While Yu et al. reported higher frequencies of ILC2s in the sputum and blood of patients with mild asthma than in those with moderate or severe asthma^31^, another study showed an increase in ILC2s in severe asthmatic patients compared to those with milder forms of asthma or healthy subjects^25^. Moreover, ILC2 proportions were increased in children with severe therapy-resistant asthma (STRA) compared with healthy controls^32^.

The responsiveness of human ILC2s to steroids and their role in steroid-resistant asthma remain a matter of debate. Peripheral IL-13-producing ILC2s have been shown to positively correlate with asthma control status^33^. Importantly, these CRTH2^+^ IL-7Rα^+^ cells are more resistant to glucocorticoids than Th2 cells^33^. In contrast, another study demonstrated that peripheral CRTH2^+^ ILC2s are inhibited by inhaled corticosteroids^34^. Similar effects were observed in ILC2s isolated from the nasal mucosa of patients with mild asthma and allergic rhinitis^35^. Moreover, Liu et al. showed that BALF ILC2s from asthmatic patients were more refractory to steroids than blood ILC2s, which was due in part to increased levels of TLSP, which conferred resistance to steroids^28^. In children with STRA, systemic but not inhaled steroids effectively reduced ILC2 numbers in sputum^36^. Recently, an inflammatory ILC2 subset that expresses CD45RO was found to be increased in patients with uncontrolled steroid-resistant asthma compared with steroid-responsive patients^37^. Further studies are warranted to address these inconsistencies, although some may be attributed to differences in disease classification criteria, patient treatment history, and the heterogeneity of human ILC2s, which we are only beginning to comprehend.

## The regulation of ILC2 function in the lungs

Recent breakthroughs in identifying various regulatory mechanisms and extracellular signals that regulate ILC2s have improved our understanding of how these cells function under steady-state and inflammatory conditions. These complex regulatory systems not only tightly control ILC2 functions but also modulate the functional plasticity and heterogeneity of these cells. Disrupting these regulatory systems results in aberrant ILC2 functions, resulting in unfavorable health issues. In this section, we provide an overview of the regulatory systems and molecules that modulate ILC2 functions in the lungs and focus mainly on studies related to airway inflammation and asthma.

### Cytokines

**Fig. 1:**
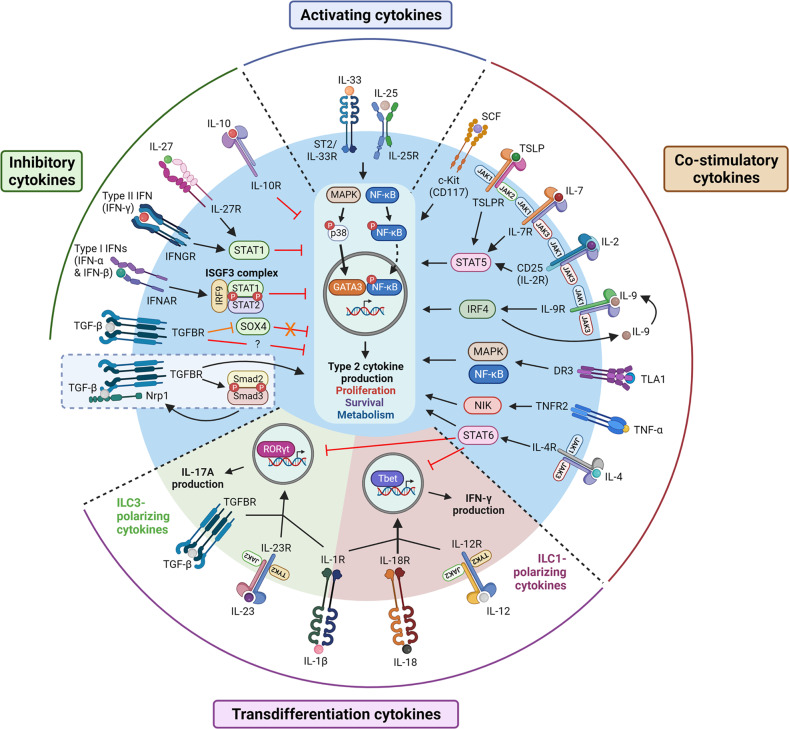
The regulation of ILC2 functions by cytokines. Activating cytokines such as IL-33 and IL-25 stimulate NF-κB translocation into the nucleus and activate the p38 MAPK pathway, resulting in GATA3 activation. Activation of NF-κB and GATA3 leads to the expression of genes involved in type 2 cytokine production, cell proliferation, survival, and metabolism. Costimulatory cytokines augment these effects in a synergistic manner. TSLP and members of the γc family of cytokines (IL-2, IL-7, IL-9, and IL-4) synergize with IL-33 through the JAK/STAT pathway, whereas TNF superfamily cytokines (TLA1 and TNF-α) act through the NF-κB pathway. Notably, IL-9 can act in an autocrine manner through IRF4. Inhibitory cytokines such as IFNs (both Type I and II) and IL-27 suppress ILC2 functions through the STAT1 pathway. The effects of TGF-β, on the other hand, are context dependent. While the mechanism underlying the suppressive effects of TGF-β is unclear, TGF-β stimulates ILC2s by promoting GATA3 expression through SOX4 inhibition (denoted in orange) or by activating the TGF-β coreceptor Nrp1 (blue dashed box). Moreover, TGF-β maintains Nrp1 expression through the canonical Smad pathway. Together with IL-1β and IL-23, TGF-β can act as a transdifferentiation cytokine that polarizes ILC2s into IL-17A-producing ILC3-like cells. Moreover, the combination of IL-1β, IL-18, and IL-12 converts ILC2s into IFN-γ-producing cells with ILC1-like properties. Notably, IL-4 inhibits ILC2 transdifferentiation. The full term for each abbreviation can be found in the text.

ILC2s respond to an array of cytokines that not only promote cell activation and function but also limit the effector response and modulate plasticity. These cytokines can be categorized into four groups: activating, costimulatory, inhibitory, and transdifferentiation cytokines^8^, as shown in Fig. 1.

#### Activating cytokines

The two main ILC2-activating cytokines are IL-33 and IL-25^8^. However, the relative heterogeneity of ILC2s in tissues affects their response to these two cytokines. Lung ILC2s express high levels of the IL-33 receptor (ST2) and therefore respond more potently to IL-33 than IL-25^7,38^. In mice, IL-33 is predominantly released by alveolar epithelial cells in response to cell injury caused by allergens such as *A. alternata* and papain^39,40^, whereas viral infection (e.g. influenza A) induces IL-33 release from alveolar macrophages (AMs)^18^. In humans, endothelial cells and bronchial epithelial cells constitutively express IL-33 and may serve as major sources during airway inflammation^41^. IL-33 stimulates ILC2 proliferation and cytokine production through the nuclear factor kappa B (NF-κB) and p38 mitogen-activated protein kinase (MAPK) signaling pathways^42,43^. IL-33-mediated lung ILC2 activation plays a crucial role in pulmonary inflammation, as evidenced by the marked reduction in eosinophilic inflammation and ILC2 activation in ST2-deficient mice compared to control mice^44^. Notably, the ability of lung ILC2s to respond to challenges in adult mice depends on prior training during the neonatal period, and this process is driven by IL-33^45^. Additionally, IL-33 is important for ILC2 trafficking from the bone marrow to the lungs^46^.

Asthmatic patients exhibit higher plasma levels of IL-25 than healthy individuals^47^. However, the correlation between this cytokine and human ILC2s in asthma remains unclear. In mice, IL-25 is predominantly produced by airway brush cells and contributes to allergen-induced experimental asthma^48^, although its effect is less potent than that of IL-33, which is partly due to its lower expression level and slower release^38^. However, intraperitoneal injection of IL-25 or helminth infection can induce the migration of intestinal ILC2s to the lungs in a sphingosine 1-phosphate-dependent manner^49^. Unlike lung-resident ILC2s, these cells exhibit an inflammatory phenotype characterized by the expression of the activation marker KLRG1 and the IL-25 receptor (IL-17RB) but not ST2 and the ability to produce IL-17A^50^. However, this phenotype is transient, and these cells eventually revert to conventional ST2^+^ lung ILC2s^50^.

#### Costimulatory cytokines

Studies have shown that the addition of IL-33 or IL-25 alone has weak effects on ILC2 activation and proliferation^51,52^, suggesting the need for additional costimulatory signals. Initial studies identified IL-2 and IL-7 as important costimulatory cytokines that augment the effects of IL-33 and IL-25. These cytokines belong to the common gamma chain (γ_c_) family of cytokines and activate the STAT5 pathway^53^. IL-2 plays a crucial role in ILC2 proliferation and survival^54^, whereas IL-7 is critical for cell development, maturation, and survival^8^. Importantly, both cytokines are cofactors that synergistically enhance IL-33-induced ILC2 activation and cytokine production^54,55^. Lung lymphatic cells have been identified as a source of IL-7, while IL-2 is mainly derived from CD4^+^ T cells, although other immune cells, such as mast cells, can also produce this cytokine under inflammatory conditions^56–58^. In addition, IL-4 and IL-9 play important roles as cofactors for ILC2 function. Similar to IL-2 and IL-7, these cytokines belong to the γ_c_ family of cytokines and can amplify cytokine production by activated ILC2s^55,59^. ILC2s can also produce IL-4 and IL-9, and the latter is produced through interferon regulatory factor 4 (IRF4)^55,60^. Autocrine IL-9 signaling supports ILC2 survival by inducing the antiapoptotic protein BCL-3^61^. While the role of autocrine IL-4 remains unclear, basophil-derived IL-4 has been shown to promote ILC2 effector functions in protease allergen-induced airway inflammation^59^. Additionally, IL-4 reverses the transdifferentiation of ILC2s into ILC1-like cells in the presence of IL-1β and IL-12^62^.

Although TSLP is often regarded as an activating cytokine, increasing evidence suggests that this factor possesses costimulatory cytokine properties with similar functions as IL-2 and IL-7^8^. First, TSLP alone does not induce cytokine production by lung ILC2s^55^ but acts in synergy with IL-33 to augment cell viability, proliferation, and cytokine production^16,55,63^. Moreover, TSLP enhances IL-33-induced IL-9 production to a level that is comparable with IL-2 and IL-7^55^. In humans, TSLP is more important for ILC2 survival than IL-33, which is required for ILC2 activation^63^. TSLP can be produced by a wide range of cells, including epithelial cells, fibroblasts, and stromal cells^64,65^. In particular, lung adventitial stromal cell-derived TSLP has been shown to support ILC2 activation and survival^65^. In addition to its role as a costimulatory molecule, TSLP confers ILC2 resistance to steroids through STAT5 signaling^22,28^.

Members of the tumor necrosis factor (TNF) superfamily have been shown to possess costimulatory functions, especially TL1A and TNF-α. TL1A is secreted by macrophages and DCs and is known to activate T cells through its cognate receptor DR3, leading to activation of the NF-κB and MAPK signaling pathways^66^. Human and mouse ILC2s express DR3, and exposure to IL-2, IL-33, and TSLP can further augment its expression by human ILC2s^30^. Moreover, TL1A is highly increased in patients with severe eosinophilic asthma compared to mild asthma^30^. Mouse studies showed that TL1A promotes ILC2 functions in synergy with IL-33 and IL-25 and mediates papain-induced ILC2 activation and the resulting lung inflammation^67,68^. TNF-α is another member of the TNF superfamily that can act as a costimulatory cytokine. Human and mouse ILC2s specifically express TNFR2, which is associated with cell survival and homeostasis, as it does not possess the death domain^69,70^. Consistent with its function, TNF-α/TNFR2 signaling enhances ILC2 activation and survival in IL-33- and *A. alternata*-challenged mice, and AMs are the predominant source of TNF-α^70^. However, unlike TL1A, TNF-α/TNFR2 signaling activates the noncanonical NF-κB pathway through NF-κB-inducing kinase (NIK)^70^.

Stem cell factor (SCF), which is the ligand for c-Kit, is important for hematopoiesis and mast cell maturation and function^71^. Serum levels of SCF and c-Kit are increased in patients with asthma and correlate with disease severity^72,73^. Although human lung ILC2s show little to no expression of c-Kit under steady-state conditions^74^, a recent mouse study demonstrated that c-Kit^+^ ILC2s accumulated in the lungs following influenza infection and contributed to eosinophil recruitment^75^. Moreover, another study showed that fibroblast-derived SCF drove c-Kit^+^ ILC2 accumulation in inflamed lungs^73^. Furthermore, SCF enhanced IL-25-mediated cytokine production in a synergistic fashion^73^, implicating its role as a costimulatory cytokine. However, the cause-and-effect relationship between SCF and human ILC2s in the context of asthma remains to be uncovered.

#### Inhibitory cytokines

ILC2 functions can be inhibited by several cytokines, such as interferons (IFNs), IL-27, IL-10, and transforming growth factor-β (TGF-β). ILC2s express IFNAR and IFNGR, which are the receptors for type I (IFN-α and IFN-β) and type II (IFN-γ) IFNs, respectively^76,77^. IFNs are produced by various immune cells, including plasmacytoid DCs, T cells, and NK cells^77–79^. In vivo studies reveal a suppressive role for IFNs in ILC2-driven airway inflammation. Indeed, exogenous administration of IFNs (IFN-β or IFN-γ) or influenza A-induced IFN-γ decreases ILC2 effector functions and ameliorates the ensuing type 2 immunopathology^78,80^. In vitro studies further show that IFNs directly inhibit ILC2 proliferation and function through the STAT1 pathway, whereas type I IFNs act through the interferon-stimulated gene-factor 3 (ISGF3) complex, which consists of STAT1, STAT2, and IRF9^78,80^. In addition to limiting cell functions, IFN-γ restricts ILC2s to the adventitia during mixed type 1-type 2 inflammation relative to type 2 inflammation, which drives ILC2 invasion in the nonadventitial parenchyma^81^. IL-27 is another cytokine that directly inhibits ILC2 functions through STAT1^78^. Consistent with its suppressive role, IL-27 deficiency exacerbates ILC2 activation in the papain model, thereby decreasing airway inflammation^82^. Similarly, interstitial macrophage-derived IL-27 was shown to attenuate ILC2-driven airway inflammation in the IL-33 model^83^.

IL-10 is an anti-inflammatory cytokine that plays a crucial role in suppressing the immune response to prevent excessive damage to the host and restore tissue homeostasis^84^. Papain- or IL-33-induced lung ILC2s express IL-10R^40^, and in vitro studies have shown that IL-10 inhibits ILC2 activation and cytokine production in humans and mice^40,85^. In contrast, IL-10 fails to suppress IL-25-activated ILC2s, suggesting specific cross-talk between the activating and inhibitory pathways in ILC2s^40,86^. Interestingly, IL-33 can also induce a subset of IL-10-producing ILC2s generated through an alternative pathway that is distinct from regulatory T cells (Tregs) and is enhanced by IL-2^87^. Specifically, these cells act in an autocrine and paracrine manner to inhibit proinflammatory ILC2 effector functions, which inhibits AHR and type 2 responses^88^.

TGF-β is another anti-inflammatory cytokine that inhibits ILC2 functions. ILC2 precursors and mature ILC2s express TGF-β receptors (TGFBR1 and TGFBR2)^89^. TGF-β is required for ILC2 development and maturation, as TGFBR2-deficient mice exhibit reduced numbers of mature ILC2s in various tissues, including the lungs^89^. However, the role of TGF-β signaling in ILC2 functions is controversial. While Treg-derived TGF-β has been shown to impair IL-13 and IL-5 production by ILC2s without affecting cell proliferation or viability^86^, pulmonary epithelial cell-derived TGF-β promotes ILC2 functions by inhibiting SRY-box transcription factor 4 (SOX4)^90^. Moreover, activation of the TGF-β coreceptor neuropilin-1 (Nrp1) by TGF-β enhances lung ILC2 function and type 2 airway inflammation by upregulating ST2 expression^91^. Notably, TGF-β maintains Nrp1 expression through the canonical Smad2/Smad3 pathway^91^. However, studies of human ILC2s revealed that TGF-β directly suppresses IL-13 and IL-5 production^85^. Collectively, these data indicate that TGF-β signaling plays a context-dependent role in regulating ILC2 activity.

#### Transdifferentiation cytokines

Various local environmental cues can influence ILC2 transdifferentiation into other ILC subsets, which is known as plasticity. Mouse and human ILC2s exhibit functional plasticity and can be converted to ILC1- or ILC3-like cells in response to appropriate cytokine combinations. IL-1β and IL-18 have been shown to polarize human ILC2s into ILC1-like cells in the presence of IL-12^92,93^. Specifically, IL-1β primes ILC2s to respond to IL-12 by inducing the IL-12 receptor subunits IL-12RB1 and IL-12RB2^93^. These ILC1-like ILC2s produce IFN-γ and exhibit increased T-bet expression, whereas GATA3 expression is decreased^62^. Notably, this conversion can be reversed by IL-4^62^. In vivo, infection with respiratory viruses (e.g., influenza A and RSV) and exposure to cigarette smoke trigger the transdifferentiation of lung ILC2s into IFN-γ-producing cells that contribute to antiviral immunity and chronic obstructive pulmonary disease exacerbation, respectively^92^. Their role in asthma development, however, remains to be determined.

Human ILC2s can acquire ILC3-like properties in the presence of IL-23, IL-1β, and TGF-β, as indicated by the induction of RORγt expression and production of IL-17A^94^. Notably, this conversion can be abolished by IL-4 or vitamin D3^94^. In mice, the conversion of lung-resident ILC2s into IL-17A-producing inflammatory ILC2s requires Notch signaling^95^. In humans, however, Notch signaling is dispensable for the conversion of blood ILC2s to ILC3-like cells^94^. These contrasting results may reflect the phenotypic differences between human and mouse ILC2s or the tissue-specific heterogeneity of ILC2s. Of note, c-Kit may influence the functional plasticity of human ILC2s, and c-Kit^hi^ ILC2s have a greater potential to convert to IL-17A-producing cells in response to ILC3-promoting cytokines than c-Kit^lo^ ILC2s^96^. This subset can also switch toward an ILC1-like phenotype in the presence of IL-12 and IL-1β^96^. Since IL-17A is involved in airway neutrophilia, the conversion of ILC2s into ILC3-like cells might contribute to neutrophilic asthma.

### Noncytokine mediators

**Fig. 2:**
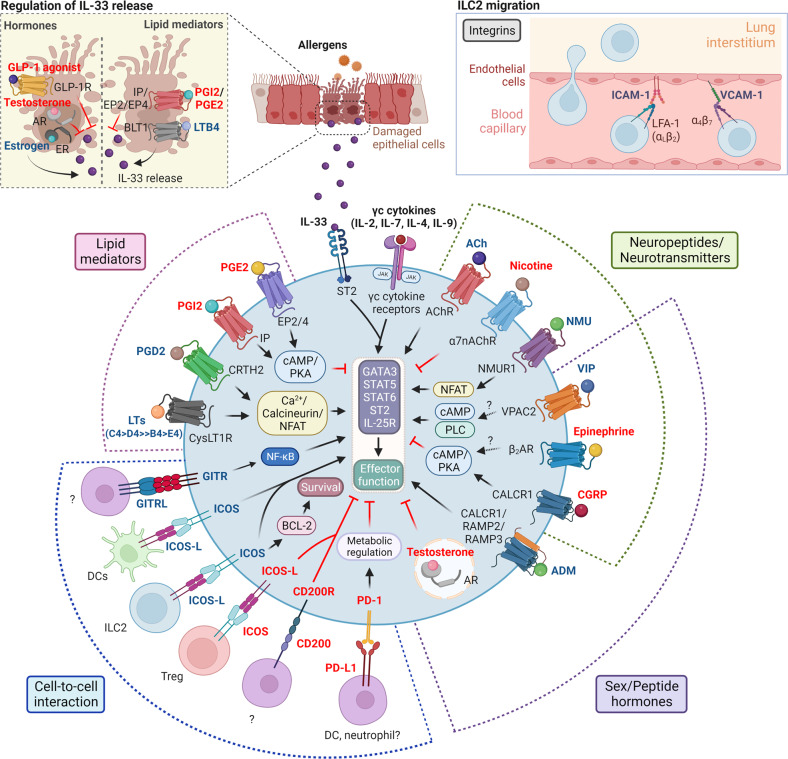
The regulation of ILC2 functions by noncytokine mediators and immunomodulatory receptors. Lipid mediators, neuropeptides/neurotransmitters, and peptide hormones are noncytokine mediators that directly regulate ILC2 functions primarily through GPCR signaling. Positive regulators (bold blue text), such as PGD2, LTs, and NMU, activate ILC2s through the Ca^2+^/calcineurin/NFAT pathway, whereas negative regulators (bold red text), including PGE2, PGI2, and CGRP, inhibit ILC2s via the cAMP/PKA pathway. VIP has been shown to activate both the cAMP and phospholipase C (PLC) pathways and may therefore promote ILC2 functions through a combination of both pathways. Downstream effects involve alterations in the expression levels of transcription factors (GATA3, STAT5, and STAT6) and surface receptors (ST2 and IL-25R) that promote ILC2 effector functions. Moreover, indirect regulation involves the induction (LTB4) or suppression (PGE2, PGI2, and GLP-1) of IL-33 production and release by damaged epithelial cells (upper left box). On the other hand, sex hormones act through nuclear receptors. While estrogen indirectly enhances ILC2 functions by stimulating IL-33 production by epithelial cells, testosterone negatively regulates ILC2 function directly and indirectly through the regulation of IL-33 expression. Interactions between ILC2s and various immune and nonimmune cells through immunomodulatory receptors such as CD200R and PD-1 result in the suppression of cell functions, whereas GITR engagement promotes ILC2 proliferation and survival through the NF-κB pathway. In addition, ILC2s express both ICOS and ICOSL. Activation of ICOS on ILC2s promotes cell activation and survival, whereas binding to ICOS on Tregs suppresses cell functions. Moreover, integrins (LFA-1 and α_4_β_7_) on ILC2s facilitate cell migration toward the lung interstitium by engaging with their respective ligands (ICAM-1 and VCAM-1) on capillary endothelial cells (upper right box). The full term for each abbreviation can be found in the text.

In addition to cytokines, ILC2s can also be regulated by noncytokine mediators such as lipid mediators, neuropeptides, and hormones that largely act through G-protein-coupled receptors (GPCRs) (Fig. 2).

#### Lipid mediators

Lipid mediators such as prostaglandins (PGs) and leukotrienes (LTs) are known to play key roles in the pathogenesis of allergic diseases, including asthma^97^. Both classes of lipid mediators are derived from arachidonic acid metabolism, which involves the cyclooxygenase and 5-lipoxygenase pathways, respectively, and bind to GPCRs^97^. Human and mouse ILC2s express receptors for various PGs and LTs, including CRTH2 (PGD receptor), E-prostanoid 2/4 (EP2/EP4; PGE2 receptor), IP (PGI2 receptor), and CysLT1R (cysteinyl leukotriene receptor)^98–101^. These lipid mediators positively or negatively regulate ILC2 activity. Positive regulation involves increasing intracellular levels of Ca^2+,^ whereas negative regulation occurs through the activation of the cyclic adenosine 3’,5’-monophosphate (cAMP)/protein kinase A (PKA) pathway^8^.

##### Prostaglandins

PGD2 is positively associated with severe asthma and is largely produced by mast cells^102,103^. The first study to show that lipid mediators could regulate ILC2 responses identified PGD2 as a positive regulator of human ILC2 functions^100^. In that study, PGD2 was shown to act in synergy with IL-33 and IL-25 to boost IL-13 function. PGD2 also serves as a chemotactic factor for human ILC2s and can upregulate the expression of IL-33 and IL-25 receptor subunits (ST2 and IL-17RA, respectively)^104^. The importance of PGD2 in ILC2 function was further demonstrated by Hardman et al., who showed that fevipiprant, a selective CRTH2 antagonist, suppressed ILC2 accumulation and type 2 cytokine production^103^. In addition, CRTH2 deficiency inhibits the accumulation of ILC2s in inflamed lung tissues in response to IL-33 treatment, indicating a role for PGD2 in ILC2 migration^105^. This effect, however, was not due to alterations in integrin or chemokine receptor expression^105^.

PGE2 and PGI2, on the other hand, negatively regulate ILC2 functions. PGE2 inhibits lung ILC2 activation by suppressing GATA3 and ST2 receptor expression via the EP4 receptor, resulting in impaired cellular activation and decreased eosinophilia and lung pathology in IL-33- and *A. alternata*-driven airway inflammation^99^. Consistent with this, a subsequent study revealed that PGE2 directly inhibited GATA3 expression, proliferation, and type 2 cytokine production in human ILC2s through EP2/EP4-cAMP signaling^106^. Mechanistically, the activation of EP2/EP4-cAMP signaling disrupts STAT5 and MYC downstream signaling pathways, impairing ILC2 energy metabolism and subsequent activation^107^. PGI2 also has a suppressive effect on ILC2 function, and mice deficient in the PGI2 receptor IP show marked increases in IL-13- and IL-5-producing lung ILC2s, leading to worsened eosinophilia and mucus production^101^. Likewise, PGI2 inhibits cytokine production by human ILC2s^101^. PGE2 and PGI2 also indirectly suppress lung ILC2 activation by inhibiting IL-33 production by epithelial cells in the *A. alternata* model^108^. In contrast, PGI2 exerts opposite effects on the HDM model, and IP-deficient mice exhibit reduced numbers of ILC2s and attenuated type 2 responses^109^. The effects seen in this model may be due to suppression by IFN-γ, since IP-deficient mice show increased numbers of IFN-γ-producing NK cells^109^. Therefore, the effects of PGI2 on lung ILC2s are largely context dependent.

##### Leukotrienes

In mice, LTB4, LTC4, LTD4, and LTE4 have been shown to positively regulate ILC2 function in a nuclear factor of activated T cells (NFAT)-dependent manner, albeit to varying degrees^110^. In particular, LTC4 and LTD4 are highly potent in stimulating cytokine production by ILC2s and have synergistic effects with IL-33^110^. A study showed that the combination of IL-33 with LTD4 or LTC4 could trigger IL-17 production by lung-resident ST2^+^ ILC2s concomitant with the expression of IL-13^111^. In addition, LTD4 can stimulate IL-4 production by ILC2s, which is a characteristic that is lacking in IL-33^98^. LTE4, on the other hand, shows different effects on mouse and human ILC2s. LTE4 alone has a weak effect on cytokine production in mice^110^ but can stimulate IL-13 production in human ILC2s^112^. Moreover, LTE4 can augment the effect of PGD2 and epithelial-derived cytokines (IL-33, IL-25, and TSLP)^112^. Notably, although LTB4 does not strongly induce ILC2 cytokine production directly^110^, it promotes ILC2 activation indirectly by stimulating IL-33 production by airway epithelial cells by engaging with its receptor BLT1^113^.

#### Neuropeptides and neurotransmitters

The respiratory tract is densely innervated by an intricate network of sensory, parasympathetic, and sympathetic neurons^114^. Recent studies have demonstrated functional cross-talk between neurons and lung ILC2s through various neuropeptides that act through GPCRs. The first neuropeptide that was shown to regulate ILC2 function was vasoactive intestinal peptide (VIP). Lung and intestinal ILC2s express the VIP receptors VPAC1 and VPAC-2^115^. VIP expression can be induced in lung afferent neurons during inflammation, as demonstrated in the ovalbumin model^116^. Importantly, blockade of VIP/VPAC-2 signaling reduced IL-13 production and ST2 expression in lung ILC2s with a concomitant decrease in type 2 inflammation^116^, suggesting that VIP positively regulates ILC2 functions. In addition to VIP, neuromedin U (NMU) also promotes ILC2 function. NMU is produced by cholinergic neurons, and lung ST2^+^ ILC2s express high levels of the NMU receptor NMUR1^117,118^. In vitro studies revealed that NMU directly promoted ILC2 function through NFAT signaling^119^. When administered intranasally, NMU alone can induce ILC2 effector functions, which culminates in airway inflammation^118^. Moreover, NMU acts synergistically with IL-25 to enhance ILC2 cytokine production, further amplifying the allergic response^117^. However, the role of NMU in allergen models remains unclear since NMUR1-deficient mice but not NMU-deficient mice fail to mount type 2 responses against HDM challenge^117^.

An initial study on calcitonin gene-related peptide (CGRP) reported that pulmonary neuroendocrine cell-derived CGRP enhances allergic responses by promoting ILC2 activation^120^. However, subsequent studies showed that CGRP limits ILC2-induced inflammation in IL-33-induced airway inflammation^121,122^. Mechanistically, CGRP induces sustained alterations in long-term gene expression by modulating chromatin regulatory elements, resulting in reduced expression of proinflammatory genes (e.g. *Il5, Il13*) and increased expression of immunoregulatory genes (e.g. *Il10ra*)^121,122^. Similarly, activation of the β2-adrenergic receptor (β_2_AR) in lung ILC2s inhibits cell proliferation and cytokine production in response to IL-33^123^. The lungs are innervated by sympathetic adrenergic neurons, which may be an endogenous source of β_2_AR ligands, such as epinephrine and norepinephrine^123^.

Acetylcholine (ACh) is a small-molecule neurotransmitter that regulates the immune system through cholinergic signaling^124^. ACh activates two types of cholinergic receptors: muscarinic and nicotinic ACh receptors^125^. Lung ILC2s express various subunits of both receptors^126^ and can produce ACh in response to *A. alternaria* challenge or alarmins such as IL-33 and IL-25^126,127^. Although neither study examined the cholinergic role of ILC2s in allergic responses, a subsequent study revealed distinct effects of ACh during allergen-induced airway inflammation^128^. While ILC2-derived ACh limits neutrophilic inflammation by reducing the expression of the neutrophil chemoattractants CXCL1 and CXCL2, non-ILC2-derived ACh enhances ILC2-driven type 2 responses and subsequent eosinophilia^128^. In contrast, activation of the α7 nicotinic acetylcholine receptor (α7nAChR) by various agonists, such as nicotine and PNU-282987, mitigates ILC2-mediated AHR and airway inflammation in the *A. alternaria* model^129,130^. Mechanistically, α7nAChR signaling inhibits ILC2 proliferation and function by inhibiting NF-κB activation and GATA3 expression^129^. These findings suggest dual roles of cholinergic signaling, which depend on the receptors being activated.

#### Hormones

Sex hormones differentially regulate ILC2 functions. In particular, lung ILC2s express the androgen receptor (AR), and testosterone and its downstream hormone 5α-dihydrotestosterone negatively regulate ILC2 proliferation and type 2 cytokine production by downregulating GATA3 and RORα expression^131^. Lung epithelial cells also express AR^132^, and testosterone suppresses IL-33 and TSLP secretion by these cells, further attenuating ILC2 proliferation and type 2 cytokine responses^131^. Notably, androgen treatment protected ovariectomized female mice from ILC2-driven airway inflammation, underscoring the therapeutic benefits of targeting AR in allergic asthma involving ILC2s^133^. Estrogen, on the other hand, plays opposing roles. Although lung ILC2s express the estrogen receptor (ER)^134^, estrogen does not have a direct effect on ILC2 function but rather stimulates IL-33 release from lung epithelial cells, which in turn enhances ILC2 activation and subsequent type 2 inflammation^135^. Overall, these findings might provide an etiological explanation for the higher numbers of ILC2s in asthmatic women and the increased prevalence of asthma in women compared to men^131,136^.

ILC2s can also be regulated by peptide hormones, including the aforementioned neuropeptides VIP, epinephrine, and CGRP. In addition, ILC2s are regulated by glucagon-like peptide-1 (GLP-1) and adrenomedullin (ADM)^137,138^. GLP-1 agonists negatively regulate lung ILC2 activation in a cell-extrinsic manner^138^. Both lung epithelial and endothelial cells express the GLP-1 receptor GLP-1R, and GLP-1R activation suppresses *A. alternata*-induced IL-33 release from damaged epithelial cells, which inhibits lung ILC2 accumulation and cytokine production^138^. In contrast, ADM directly promotes IL-5 production by lung ILC2s in a synergistic manner with IL-33^137^. In vivo, ADM is released by lung epithelial cells during hypoxia and contributes to ILC2 accumulation in the lungs and airway eosinophilia in mice challenged with papain^137^.

### Cell-to-cell signaling

In addition to indirect regulation by mediators released from various cell types, pulmonary ILC2s are also regulated through ligand-receptor interactions with immune and nonimmune cells (Fig. 2). In particular, inducible T-cell costimulator (ICOS) and its ligand ICOSL are expressed on human and mouse ILC2s^139^, although the effect of ICOS signaling is context-dependent. Induced Tregs, which express ICOS, inhibit lung ILC2 functions through engagement with ICOSL on ILC2s^86^. In contrast, the ICOS:ICOSL interaction between ILC2s promotes survival and cytokine production, resulting in airway inflammation and AHR^139^. Likewise, the binding of ICOSL on DCs to ICOS on ILC2s enhances lung ILC2 activity and pulmonary inflammation in the papain model^140^.

Activated lung ILC2s exhibit increased expression of various immunoregulatory receptors, such as programmed death-1 (PD-1), CD200R, cytotoxic T-lymphocyte associated protein 4 (CTLA-4), T-cell immunoreceptor with Ig and ITIM domains (TIGIT), and glucocorticoid-induced TNFR related protein (GITR)^141,142^. PD-1 serves as an important metabolic checkpoint to keep ILC2 effector functions in check during inflammation, and blocking PD-1 signaling on ILC2s exaggerates cell activity and exacerbates AHR^143^. Likewise, CD200R engagement on ILC2s inhibits cell activation, proliferation, and type 2 cytokine production^144^. While neutrophils and DCs are likely sources of the PD-1 ligand in IL-33-dependent inflammation^143^, the CD200R ligand (CD200) is upregulated on multiple pulmonary cell types, including fibroblasts and T cells, in asthmatic patients^144^. The exact cell type that engages CD200R on ILC2s remains to be determined. IL-33-activated lung ILC2s upregulate the coinhibitory molecules CTLA-4 and TIGIT^87^, and their ligands are typically expressed on antigen-presenting cells^145,146^. Given the inhibitory effects of these molecules on NK cell and T-cell functions^147^, engagement of these receptors may also inhibit ILC2 functions. Moreover, engagement of GITR by GITRL, which is expressed on endothelial cells, myeloid cells, and B cells^148^, promotes lung ILC2 proliferation and survival, and GITR deficiency impairs ILC2-driven lung inflammation in the papain model^149^. Likewise, GITR engagement augments cytokine production by IL-33-activated human ILC2s^142^.

ILC2 migration from the circulation to the lung during inflammation is facilitated by interactions with endothelial cells through adhesion molecules such as integrins. Lung endothelial cells from asthmatic patients express high levels of the adhesion molecules intracellular adhesion molecule-1 (ICAM-1) and vascular cell adhesion molecule-1 (VCAM-1)^150^. Human and mouse lung ILC2s express LFA-1 (α_L_β_2_) integrin, the receptor for ICAM-1^151^. An initial study suggested that β_2_ integrin was crucial for ILC2 trafficking from the circulation in response to *A. alternata* challenge^151^. Subsequent studies further revealed distinct roles for LFA-1 and ICAM-1. LFA-1 was shown to regulate ILC2 migration to the lungs, whereas ICAM-1 is required for ILC2 development and function^152,153^. In addition, IL-33 is a potent inducer of VCAM-1 expression on lung endothelial cells through receptors for advanced glycation end products (RAGE) signaling^154^. VCAM-1 mediates ILC2 recruitment to the lungs through β_7_ integrin, as evidenced by the failure to induce lung ILC2 accumulation and type 2 inflammatory responses in mice treated with anti-VCAM-1 or anti-β_7_-integrin blocking antibodies^154^.

### Metabolic regulation

**Fig. 3:**
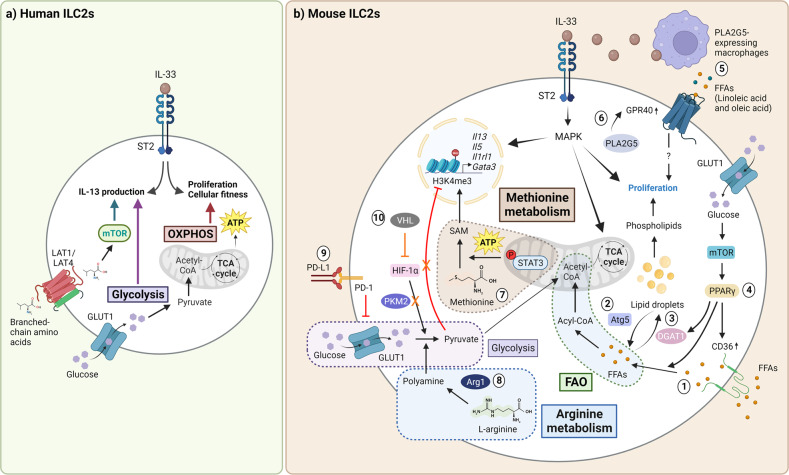
The metabolic reprogramming of activated ILC2s in humans and mice. **a** IL-33-activated human ILC2s rely on OXPHOS to sustain fitness and proliferation, whereas IL-13 production is dependent on glycolysis and the mTOR pathway. **b** In mice, activated ILC2s rely primarily on FAO (green dashed region) and amino acid metabolism. FFAs are acquired externally through CD36 (**1**) and are temporarily stored as lipid droplets, which are regulated by Atg5-dependent autophagy (**2**) and DGAT1 (**3**). PPAR-γ regulates lipid uptake by promoting CD36 and DGAT1 expression, whereas its expression is regulated by glucose/mTOR signaling (**4**). Fatty acids stored as lipid droplets are further used to fuel FAO or converted into phospholipids to promote ILC2 proliferation. PLA2G5-expressing macrophages are important sources of FFAs (namely, linoleic acid and oleic acid) under inflammatory conditions (**5**). Moreover, PLA2G5 intrinsically regulates GPR40 receptor expression on ILC2s, and activation of this receptor by FFAs, particularly linoleic acid, promotes ILC2 expansion through an undefined mechanism (**6**). Amino acids such as methionine and arginine play crucial yet distinct roles in ILC2 functions. Methionine metabolism (brown dashed region) epigenetically regulates *Il13* and *Il5* by producing the methyl-donating substrate SAM through a STAT3-dependent mechanism (**7**). In contrast, Arg1 metabolizes arginine to fuel polyamine biosynthesis (blue dashed region), which supports glycolysis (**8**). Functional fine-tuning of glycolysis (purple dashed region) is important to limit ILC2 functions. PD-1 is an important metabolic checkpoint that keeps glycolysis in check, and PD-1-deficient ILC2s exhibit increased glycolysis and aberrant functions (**9**). Moreover, the PKM2/pyruvate checkpoint, which negatively regulates *Il1rl1* (which encodes ST2) expression by suppressing H3K4me3, is kept in check by the E3 ubiquitin ligase VHL through HIF-1α degradation (**10**). The effects of VHL are denoted in orange. The full term for each abbreviation can be found in the text.

Similar to T cells, ILC2s undergo metabolic reprogramming to meet the energetic demands imposed by activation, expansion, and effector functions (Fig. 3). In the resting state, ILC2s rely on oxidative phosphorylation (OXPHOS) to sustain homeostatic functions^155^. During activation, human ILC2 proliferation and effector functions are metabolically uncoupled, and OXPHOS plays an important role in maintaining cellular fitness and proliferation, whereas glycolysis and the mammalian target of rapamycin (mTOR) pathway regulate cytokine production^156^. In mice, the pathogenic role of lung ILC2s is dependent on fatty acid oxidation (FAO) and lipid droplet formation^157,158^. The ability of ILC2s to use FAO depends on autophagy, and Atg5 deficiency impairs FAO, resulting in impaired proliferation and death by apoptosis^157^. Moreover, the formation of lipid droplets is important for the production of phospholipids that fuel ILC2 proliferation and is facilitated by glucose^158^. Glucose mediates this process by regulating the gene expression of diglyceride acyltransferase (DGAT1) and peroxisome proliferator-activated receptor gamma (PPARγ) through the mTOR pathway^158^. Together with CD36, PPARγ promotes lipid uptake^159^. Group V phospholipase A_2_ (PLA2G5)-expressing macrophages have been shown to serve as a source of free fatty acids (FFAs) during inflammation^160^. Moreover, PLA2G5 plays an intrinsic role in regulating IL-33 release from macrophages and the expression of the FFA receptor GPR40 on ILC2s^160^.

Amino acid metabolism is essential for lung ILC2 activation and function. In particular, arginine metabolism by arginase 1 (Arg1) is required for allergen-induced ILC2 proliferation and cytokine production, as evidenced by the lack of airway inflammation in Arg1-deficient mice due to impaired ILC2 effector functions^161^. Further supporting this is the observation that inhibiting SLC7A8, a crucial amino acid transporter for arginine and large amino acids, reduces ILC2 frequency and cytokine-producing capacity, resulting in impaired eosinophil recruitment to the lungs in response to HDM challenge^162^. Notably, methionine metabolism promotes ILC2-driven airway inflammation through STAT3-dependent production of S-adenosylmethionine (SAM), which epigenetically regulates the expression of IL-13 and IL-5^163^.

In general, dysregulation of glycolysis causes aberrant ILC2 functions. A study by Helou et al. described PD-1 as a metabolic checkpoint that regulates ILC2 effector functions^143^. In the absence of PD-1, ILC2s undergo metabolic reprogramming in favor of glycolysis in response to activation by allergens or IL-33, resulting in enhanced ILC2 functions and the exacerbation of airway type 2 responses^143^. In a separate study, the E3 ubiquitin ligase VHL was reported to play a crucial role in late-stage maturation and effector functions of ILC2s by inhibiting the hypoxia-inducible factor 1α (HIF-1α)/glycolysis axis^164^. VHL deficiency increases glycolysis and pyruvate kinase M2 (PKM2) expression, which downregulates ST2 expression^164^. Therefore, fine-tuning essential metabolic pathways is pertinent to maintaining lung ILC2 homeostasis and regulating their effector functions.

### ILC2-regulating metabolites

**Fig. 4:**
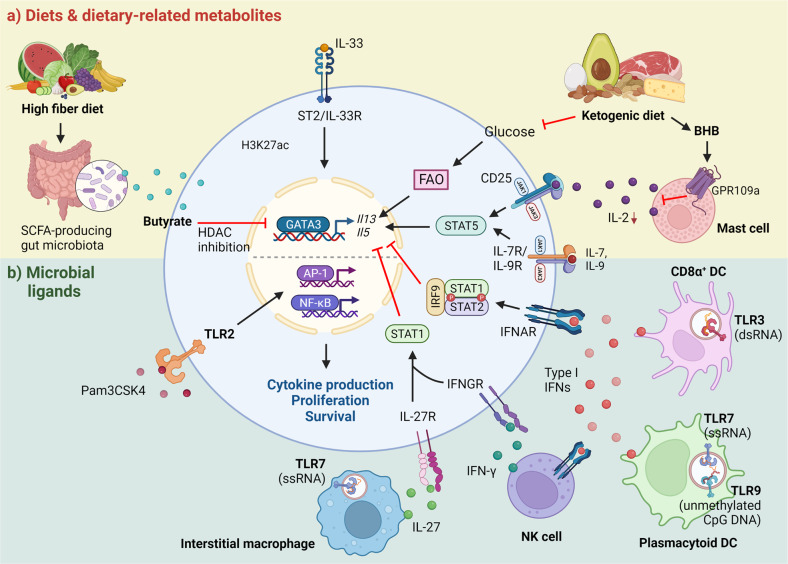
The influence of diet/diet-derived metabolites and microbial ligands on ILC2 activity. **a** A fiber-rich diet can increase SCFA-producing gut microbes. In particular, the SCFA butyrate negatively regulates ILC2 functions through HDAC inhibition, which suppresses the expression of GATA3 and its downstream genes, including *Il13* and *Il5*. The glucose-deprived ketogenic diet attenuates ILC2 functions by inhibiting FAO (see Fig. 3, pathways 3 and 4 for a detailed mechanism). The ketogenic diet also increases the ketone body BHB. BHB can attenuate ILC2 functions by suppressing IL-2 production by mast cells via GPR109a. **b** Exposure to microbial ligands activates TLRs, which can directly or indirectly influence lung ILC2 activity. TLR2 acts directly on ILC2s to stimulate cytokine production by activating the transcription factors AP-1 and NF-κB. In contrast, activation of endosomal TLRs (TLR3, TLR7, and TLR9) on myeloid cells negatively regulates ILC2 functions through IFNs and IL-27 in a STAT1-dependent manner. The activation of TLR3 and TLR7 on CD8α^+^ DCs and plasmacytoid DCs, respectively, induces type I IFN production, which inhibits ILC2s through IFNAR signaling. Plasmacytoid DCs also express TLR9, and the activation of TLR9 by unmethylated CpG DNA triggers type I IFN production, which in turn stimulates IFN-γ-producing NK cells to suppress ILC2s through IFNGR. Moreover, TLR7 activation on interstitial macrophages induces IL-27 production, which negatively regulates ILC2s via IL-27R signaling. The full term for each abbreviation can be found in the text.

In this section, we will discuss the roles of microbial products/ligands and dietary metabolites that have been shown to regulate lung ILC2 function directly or indirectly (Fig. 4).

#### Microbial products and ligands

An environment rich in microbes has been linked to lower rates of asthma^165^, prompting studies to investigate the effects of microbial infection or exposure to microbial ligands. The activation of endosomal Toll-like receptors (TLR3, TLR7, and TLR9) in myeloid cells by nucleic acid analogs that mimic viral RNA or bacterial DNA limits lung ILC2 activation through the production of IFNs or IL-27^83,166,167^. Conversely, direct activation of TLR2 by its ligand Pam3CSK4 induces IL-13 and IL-5 production by ILC2s through NF-κB and AP-1 signaling^168^. Moreover, the effect of Pam3CSK4 is enhanced by HDM stimulation, as evidenced by heightened type 2 inflammatory responses and AHR^168^. While these studies focused on the short-term effects of pathogen infection or exposure to microbial ligands, a recent study by Block et al. elegantly demonstrated the impact of physiologically acquired infections on allergen-mediated inflammation by cohousing laboratory mice with pet store mice^169^. The results showed that the limiting effects of microbes on lung ILC2 responses to *A. alternata* challenge are transient, and long-term cohousing did not inhibit ILC2-driven type 2 responses^169^. Overall, while most of these studies suggest that microbial components protect against allergen-induced type 2 inflammation, recent exposure rather than cumulative exposure dictates responsiveness to allergens.

#### Diets

It is well established that our dietary habits can influence asthma outcomes and risks. Indeed, diets rich in saturated fat are associated with an increased risk of asthma symptoms^170^. Moreover, a fiber-rich diet decreases Th2 cell-mediated allergic inflammation through gut microbiota-derived short-chain fatty acids (SCFAs)^171^. Subsequent studies on ILC2-driven AHR and type 2 inflammation showed similar results. Specifically, butyrate was shown to inhibit human and mouse ILC2 proliferation and cytokine production by inhibiting histone deacetylases (HDACs), which reduced GATA3 expression^172,173^. In addition to a high-fiber diet, a ketogenic diet (KD) modulates ILC2 functions by regulating lipid metabolism^158^. Restricting glucose intake through the KD inhibits fatty acid metabolism and lipid droplet formation in ILC2s, resulting in impaired activation^158^. Additionally, beta-hydroxybutyrate (BHB) is one of the ketone bodies that is increased during the KD^174^. Our recent study indicated that BHB indirectly suppressed ILC2-driven AHR and airway inflammation by inhibiting IL-2 production by lung mast cells partly through GPR109a signaling^56^. Our findings highlight the potential benefits of ketone body supplementation to manage asthma.

## Concluding remarks

In this review, we highlighted the current understanding of the role of ILC2s in asthma based on results from experimental mouse models and clinical studies. We also provided a comprehensive overview of various regulatory factors that have been identified as critical modulators of lung ILC2 activity. Understanding how these factors regulate ILC2 effector function is crucial for controlling their activity under inflammatory conditions, as aberrant activation is a cause of dysregulated type 2 responses in allergic asthma. Furthermore, identifying regulatory mediators that can switch ILC2s to other nontype 2 cytokine-producing ILC-like cells may provide important clues to understand the contribution of these cells to nontype 2 asthma phenotypes.

Despite this progress, there are many unanswered questions pertaining to the biology of ILC2s and their role in asthma pathogenesis. First, research on ILC2 heterogeneity is still in its infancy. While mouse ILC2s can generally be categorized into two subtypes (natural and inflammatory ILC2s), at least five different subtypes have been identified in humans. Whether more functional subtypes exist remains to be determined. Additionally, whether and how different subtypes contribute to various asthma endotypes warrants further investigation. This biological complexity also raises the question of whether it is possible to develop effective asthma treatments by targeting ILC2s. Furthermore, while the role of ILC2s as effector cells and orchestrators of downstream inflammatory responses has been established in mice, the understanding of ILC2-immune cell interactions in humans remains incomplete. Therefore, further studies to bridge the gap in knowledge between mice and humans will facilitate the translation of preclinical results into the clinical setting for the management or control of asthma.
